# Gender Norms and HIV Testing/Treatment Uptake: Evidence from a Large Population-Based Sample in South Africa

**DOI:** 10.1007/s10461-019-02603-8

**Published:** 2019-07-29

**Authors:** J. Pulerwitz, A. Gottert, K. Kahn, N. Haberland, A. Julien, A. Selin, R. Twine, D. Peacock, X. Gómez-Olivé, S. A. Lippman, A. Pettifor

**Affiliations:** 1 HIV and AIDS Program, Population Council, Washington, DC USA; 2 MRC/Wits Rural Public Health and Health Transitions Research Unit (Agincourt), School of Public Health, Faculty of Health Sciences, University of the Witwatersrand, Johannesburg, South Africa; 3 Poverty, Gender, and Youth Program, Population Council, New York, USA; 4 Department of Epidemiology, University of North Carolina at Chapel Hill, Chapel Hill, USA; 5 Promundo-US, Washington, DC USA; 6 Division of Prevention Science, Department of Medicine, University of California San Francisco, San Francisco, USA; 7 Population Council, 4301 Connecticut Avenue, NW, Suite 280, Washington, DC 20008 USA

**Keywords:** Gender norms, HIV testing, Antiretroviral treatment

## Abstract

How does the endorsement of different dimensions of gender norms by men and/or women influence their use of HIV testing and antiretroviral treatment? This question was examined using data from a 2014 population-based survey of 1053 women and 1004 men, ages 18–49, in rural South Africa. We used a global measure for views toward gender norms (the GEM Scale), plus four subsets of scale items (all reliabilities ≥ 0.7). In multivariate analyses using the global measure, endorsement of inequitable gender norms was associated with more testing (AOR 2.47, p < 0.01) and less treatment use (AOR 0.15, p < 0.01) among women but not men. When examining specific subsets of inequitable norms (e.g., endorsing men as the primary decision-maker), decreased odds of treatment use was found for men as well (AOR 0.18, p < 0.01). Careful attention to the role specific gender norms play in HIV service uptake can yield useful programmatic recommendations.

## Resumen

¿Cómo la aprobación de diferente dimensiones de normas de género entre hombres y mujeres influencian su utilización del examen del VIH y el tratamiento antiretroviral? Esta pregunta fue examinada mediante el uso de datos de una encuesta de 1053 mujeres y 1004 hombres, de edades entre 18 y 49 años, en áreas rurales de Sudáfrica en el 2014. Este estudio usó una medida global por atitudes hacia género (la escala GEM, en sus siglas en inglés), y cuatro ítems de escala (con confianza ≥ 0.7). En el análisis multivariable que utiliza la medida global, la aprobación de normas de inequidad de género está asociada con mayores probabilidades de exámenes (AOR 2.47, p < 0.01) y menor tratamiento (AOR 0.15, p < 0.01) entre las mujeres pero no entre los hombres. Al examinar conjuntos específicos de normas de inequidad (por ejemplo, apoyo a la idea que los hombres son los principales tomadores de decisión), disminuyen las probabilidades de uso de tratamiento entre hombres (AOR 0.18, p < 0.01). La atención al rol de las normas de género en la utilización de servicios de VIH puede ofrecer recomendaciones útiles para los programas.

## Introduction

Substantial global evidence has demonstrated that endorsement of inequitable gender norms is linked with a series of negative health outcomes related to HIV/STI risk and prevention [1–11]. These inequitable gender norms include, for example, that men should make all the major decisions in the household; women are solely responsible for pregnancy prevention; and a husband has the right to be physically violent with his wife if she does not obey him. Studies have shown associations between agreement with inequitable norms and having multiple sexual partners [6, 7, 11]; intimate partner violence (IPV) [1–7, 10, 11]; less condom use [3, 4, 6–9]; and early sexual debut [10]. A systematic review to assess the efficacy of HIV violence and prevention programs for men that attempted to promote more equitable gender norms (especially those around masculinity) found that these programs can improve protective sexual behaviors and reduce HIV and STI risk [12].

Comparatively little is known about the role of gender norms in the HIV care continuum, including HIV testing [13], and care and treatment for people living with HIV [14]. In the current era of early and universal HIV treatment [15], and the global promotion of HIV testing as an entry-point to care, it is critical to understand this relationship [16–19]. Clarifying how inequitable gender norms may act as a barrier to being linked to, or staying engaged in, HIV care and treatment, for example, will both help us to meet the HIV care needs of individual women and men, as well as achieve more public health-oriented HIV epidemic control goals.

Further, there are a number of different dimensions of gender norms and a more nuanced understanding of which norms are most associated with which key health and service use outcomes is needed. For example, it may well be that certain norms influence HIV prevention behaviors like condom use while others influence adherence to antiretroviral (ART) medications. Related findings would help suggest the programmatic focus of future programs.

While there is a growing body of research on gender norms and how they are linked with HIV service use and care outcomes, most of the investigations have been on a small scale and have been largely qualitative. A systematic review focused on gender norms/masculinity and men’s HIV testing found that out of 642 studies examined, only 14 met the inclusion criteria and all were qualitative [13]. Findings, however, suggested that gender-related themes play an important role. Examples of such themes included that discussion of HIV testing with partners was viewed as a threat to men’s authority, that changing sexual risk behavior may undermine men’s masculine identity, and that seeking help from others was associated with weakness. Studies that have explored the influence of gender and partner relationship dynamics on HIV care/treatment have also highlighted the connection. For example, studies with Ugandan and Malawian women living with HIV who had not entered care/treatment after HIV diagnosis found that lack of support from a male partner was a major reason for this [20, 21].

South Africa has the largest HIV epidemic in the world, with an estimated seven million people living with HIV [22]. The South African government adopted the 90-90-90 targets in 2015 as part of efforts to end the AIDS epidemic [23], and it currently has the largest HIV treatment program in the world, with over half of adults with HIV on ART [24]. Gender-based violence is also endemic. According to South Africa’s 2016 Demographic and Health Survey (DHS), one in five women older than 18 has experienced physical violence [25], and another study reported that three women die at the hands of their intimate partner every day [26]. Gender dynamics such as those related to violence have been identified as a major contributor to the HIV epidemic [14].

Given evidence to date, there is a need to quantitatively examine the role of gender norms in influencing HIV testing and treatment use with a large sample of participants. There is also a need to “unpack” specific gender norms by topic area so that the salience of different dimensions of gender norms can be understood, as well as their associations with key outcomes. To this end, we explored these issues using data from a large population-based sample in Mpumalanga, South Africa. We first describe women’s and men’s attitudes toward a variety of gender norms, then discuss associations with HIV testing and treatment uptake.

## Methods

### Data Source and Study Setting

Data are drawn from a population-based survey of 2057 individuals (1053 women and 1004 men) ages 18–49 in 27 villages in the rural Bushbuckridge sub-district of Mpumalanga province. This survey served as an endline assessment for a community-based trial (Community Mobilization for the Prevention of HIV in Young South African Women) funded by the United States National Institute for Mental Health (NIMH) and as a baseline assessment for another NIMH-funded trial (Community Mobilization for Treatment as Prevention) [27, 28]. The villages in the study are part of the Agincourt Health and Socio-demographic Surveillance System (Agincourt HDSS) run by the Medical Research Council/Wits University Rural Public Health and Health Transitions Research Unit, where an annual census has been conducted since 1992 [29].

Bushbuckridge sub-district, like much of the province, is characterized by high levels of poverty and migration, low levels of education, and few employment opportunities. Mpumalanga has the second-highest adult HIV prevalence of South Africa’s nine provinces, at 22% [30].

### Sample

The sampling frame consisted of all HDSS households with a resident aged 18–49 enumerated during the 2013 Agincourt HDSS update [29]. We sampled either a man or woman from each household based on HDSS data (in order to generate adequate samples for both men and women in each community). Individuals in the household were randomly ranked (1, 2, 3, etc.). Upon entering a home, the individual randomly ranked first was screened for the following more detailed eligibility criteria: person lived in the home, aged 18–49 per confirmed date of birth, and had lived in the study area for most of the past 12 months. If the first individual did not meet these eligibility criteria, the second was screened, and so on. Only one individual was interviewed per household.

The initial sampling frame included 3456 households across 27 villages, of which 3061 (88.6%) were contacted. Eligibility could not be determined for 52 of these households. For 939 households, the selected individual was not eligible (often due to not meeting residency criteria). In total, 2070 screened individuals were eligible to participate, with 2057 (99%) consenting to participate.

### Survey Procedures

Surveys took place at the participant’s house, and generally were one to two hours in length. They were conducted by a trained interviewer in the local language of Shangaan or in English, depending on the respondent’s preference. Surveys were administered using computer assisted personal interviewing (CAPI), in which the interviewer reads each question to the respondent, then enters the answer into an electronic form on a laptop computer.

### Measures

#### Views Toward Gender Norms

We measured views toward gender norms with the Gender Equitable Men (GEM) Scale. The GEM Scale was originally developed by Pulerwitz and Barker (2008) in Brazil [3] and has now been used in many studies of HIV risk and violence behaviors in sub-Saharan Africa, among both men and women [5, 7, 31]. A set of 40 items representing inequitable gender norms was included in the survey (both original GEM Scale items and about 15 new items developed for this study). Response categories were “Do not agree at all,” “Somewhat agree,” and “Agree a lot.” All factor and reliability analyses were carried out in Stata v15.0 [32].

From amongst the 40 items, we selected sets of items that, based on the literature, reflected four gender norms dimensions hypothesized to be most relevant to HIV service use. The final dimensions and composite scale items are shown in Table 2. These included norms condoning men’s violence and control over women (7 items); norms around men as the decision-maker in a couple (6 items); norms around men’s toughness and avoidance of help-seeking (5 items); and norms around women’s primary responsibility as family caretaker (5 items). All dimensions were contained in the original GEM Scale, except for items related to men’s avoidance of help-seeking. To arrive at each final set of items, we removed one to two originally-included items based on low factor loadings (< 0.30). Finally, we also constructed a ‘composite,’ adapted GEM Scale variable comprised of all items across the dimensions (23 items).

We generated aggregate scores for each individual on the GEM scale-composite and the four dimensions addressed in the scale by taking the mean of non-missing items and multiplying by the number of items in the set. Higher scores represent more inequitable norms on the GEM Scale-composite and the four included dimensions. As shown in Table 2, the composite scale as well as each dimension had good internal reliability, with all Ordinal Thetas ≥ 0.70. (Theta is a measure of reliability similar to Cronbach’s coefficient alpha but more suitable for categorical response categories [33, 34]). Each GEM Scale dimension also had good model fit in confirmatory factor analyses, based on commonly recommended cut-off criteria [33].

#### HIV Testing in the Last 12 Months

Participants were asked “In the past 12 months, how many times have you been tested for HIV?”. We created a binary testing variable (i.e., at least one test versus zero tests in the last 12 months), and recoded as missing any individuals who reported being HIV positive for over one year (n = 54 women and 17 men).

#### Current ART Use

Participants who reported being HIV positive (i.e., answered ‘HIV positive’ in response to the question “What were the results of your last HIV test, or the last test for which you received results)?”, were considered as currently using ART if they answered ‘yes’ to both of the following questions: “Are you on antiretroviral treatment or ART now or were you ever on ART?” and “Are you still taking ART?”.

### Statistical Analysis

Using Stata v15.0 [32], we generated weighted means and proportions for variables of interest, and conducted weighted logistic regression to examine associations of the GEM Scale-composite and included dimensions with last-year HIV testing and current ART use. We used separate models for women and men. Scaled weights, determined based on the proportion of total eligible households per village and total eligible women or men per household, were used to account for differential sampling probabilities and to represent the distribution of women and men aged 18–49 years in Agincourt based on the 2014 Agincourt HDSS. Robust standard errors were used to account for clustering by village [32].

The study was approved by the institutional review boards at the University of California-San Francisco, the University of North Carolina at Chapel Hill, the Human Research Ethics Committee at the University of the Witwatersrand in South Africa, and the Mpumalanga Department of Health and Social Development Research Committee.

## Results

Sociodemographic characteristics of participants are presented in Table 1. The mean age was 31.5 years for women and 29.4 for men. About 39% of women, and 23% of men, reported being married. About one-third of both women and men said they had completed high school, and about one-third had received any income in the past three months.

**Table 1:** Sociodemographic characteristics of participants

	Women (n = 1015)	Men (n = 1004)
Age (mean, range)	31.5, 18–49	29.4, 18–49
Married (vs. other)	39.1%	22.8%
Completed high school	31.8%	30.6%
Received any income in the past 3 months	36.4%	31.8%

Analyses incorporated sampling weights and accounted for clustering

### Profiling Dimensions of Gender Norms

Percentages of respondents who “agree a lot” or “somewhat agree” (vs. “do not agree at all”) with each GEM Scale item are presented in Table 2. We collapsed the “agree” response categories for ease of interpretation; for most items fewer than 10% of respondents answered “somewhat agree.” Also included in Table 2 are mean scores for the composite variable as well as the four included dimensions.

**Table 2:** Gender norms among women and men (n = 1053 women; 1004 men)

	% who “agree a lot” or “somewhat agree” with statement (vs. “do not agree at all”)	
	Women	Men
GEMS composite (combining 4 sub-dimensions below) (λ = 0.88) Higher = more inequitabl*e*—mean (range 1–3)	1.81	1.87*
Norms condoning men’s violence and control over women (λ = 0.76) Mean	1.76	1.87*
A man is expected to discipline his woman	73.5	75.5
Sometimes a man needs to put a woman in her place	67.6	68.8
A woman who is unfaithful needs to be put in her place	56.0	59.9
A woman should obey her husband in all things	46.3	50.2
A man using violence against his wife is a private matter that shouldn’t be discussed outside the couple	36.1	43.7
There are times when a woman deserves to be beaten	10.3	24.0
A man can hit his wife if she won’t have sex with him	8.3	7.8
Norms around men as the decision-maker in a couple (λ = 0.70) Mean (range 1–3)	1.80	1.90*
A man should have the final word about decisions in his home	67.0	73.1
The husband should decide to buy the major household items	59.2	64.8
If a woman says no to sex, she usually doesn’t mean it	63.3	67.8
You don’t talk about sex, you just do it	24.7	29.4
It is the man who decides what type of sex to have	22.7	30.8
A man should be outraged if his wife/partners ask him to use a condom	27.2	29.4
Norms around men’s toughness and avoidance of help-seeking (λ = 0.79) Mean (range 1–3)	1.49	1.53
To be a man, you need to be tough	51.0	54.9
If someone insults a man, he should defend his reputation with force if he has to	33.4	33.1
For men, getting sick is a sign of weakness	26.8	25.9
A man shouldn’t go to the doctor unless his situation is serious	13.2	17.6
Health clinics are for women and children	15.6	15.9
Norms around women’s primary responsibility as family caretaker (λ = 0.71) mean (range 1–3)	2.20	2.20
It is a woman’s responsibility to avoid getting pregnant	85.4	77.3
A woman’s role is taking care of her home and family	76.7	78.2
Changing diapers, giving a bath, and feeding kids are the mother’s responsibility	65.9	67.4
A woman should tolerate violence to keep her family together	43.6	48.1
Only when a woman has a child is she a real woman	41.5	44.8

Analyses incorporated sampling weights and accounted for clustering

λ = Ordinal theta (measure of internal consistency reliability similar to Cronbach’s alpha)

*p < 0.001. *p* value is for the difference in mean score between women and men, from weighted bivariate analyses that accounted for clustering

Most GEM Scale items received endorsement from a relatively high proportion of both men and women, indicating general agreement with inequitable norms. Mean levels of the composite GEM Scale score and norm domains fell close to the middle of the range of 1.0 to 3.0 (higher = more inequitable). The most gender-inequitable domain was related to women’s primary responsibility as family caretaker. Views that men should be the primary decision-maker in a couple were also quite inequitable, particularly around major household decisions (e.g., “A man should have the final word about decisions in his home”.) Within the domain concerning men’s violence and control over women, respondents expressed the most support for the statement that “A man is expected to discipline his woman” rather than an explicit statement around violence such as “There are times when a woman deserves to be beaten.” The exception to the high support for inequitable gender norms relates to the new domain around men’s toughness and avoidance of help-seeking. In particular, respondents reported very low agreement with items endorsing the view that men shouldn’t seek health care (e.g., “A man shouldn’t go to the doctor unless his situation is serious”) but they did still maintain some agreement with the norm that men should be tough.

Comparing levels of support for inequitable norms among women and men, in general they appeared similar across all norm domains. However, these relatively small differences were statistically significant in some cases. That is, men held significantly more inequitable views than women using the GEM Scale composite, as well as the domains around men’s violence and control over women, and men as the decision-maker in a couple. There was no difference in support for men’s toughness and avoidance of help-seeking or women’s primary responsibility as family caretaker.

Regarding differences by age/age group, endorsement of gender norms was also quite similar across age groupings (results not shown). However, while there were no statistically significant differences among men, there were a few among women. That is, views toward the overall GEM Scale composite and norms around men as primary decision-makers in a couple, became increasingly inequitable with women’s increasing age. For example, among women, the mean GEM Scale composite score rose from 1.76 to 1.82 to 1.83 among women ages 18–24, 25–35, and 36–49, respectively (p < 0.01).

### Profiling HIV Service Use

HIV service use behaviors are shown in Table 3. Nearly all women, and about three-quarters of men, reported ever being tested for HIV. Within the last 12 months, 78.9% of women reported having tested for HIV, versus 54.9% of men. In addition, about half of men and women reported talking with their current/most recent partner about getting tested; this was slightly higher among women than men (56.4% vs. 46.5%).

**Table 3:** HIV service use among women (n = 1053) and men (n = 1004) by age

	Women aged%				Men aged%			
	18–24	25–35	36–49	Total	18–24	25–35	36–49	Total
Ever tested	94.5	97.3	94.4	95.6	78.2	75.4	78.7	77.6
Tested in last 12 months	79.8	84.1	71.3	78.9	54.0	54.3	56.6	54.9
Talked with current/most recent sexual partner about getting tested for HIV	58.4	57.4	53.5	56.4	42.9	54.3	44.9	46.5

Among HIV positive	–	–	–	(n = 122)	–	–	–	(n = 48)
Ever taken ART	–	–	–	72.5	–	–	–	83.0
Currently taking ART	–	–	–	70.8	–	–	–	78.7

Analyses incorporated sampling weights and accounted for clustering. For ART use, we chose to report only the total percentages because sample sizes were too small for some age categories

Turning to use of ART among respondents reporting that they were HIV positive (n = 122 women, 48 men), nearly three-quarters of women reported ever taking ART, and currently taking ART (72.5% and 70.8%, respectively). Among men, reported ART use was slightly higher with 83.0% ever having taken ART, and 78.7% currently taking ART.

### Associations Between Gender Norms and HIV Testing

Multivariate analysis results for HIV testing in the last year are presented in Table 4. Among women, endorsing more inequitable norms in general (GEM Scale composite), and particularly women’s primary responsibility as family caretaker, were associated with increased odds of testing in the last year. Findings from ancillary analyses among women suggest that this association may be accounted for by greater likelihood of testing after having children/during pregnancy. When controlling for other demographic characteristics, having biological children (a possible proxy for recent pregnancy, a variable not included in the survey) was significantly associated with endorsing norms around women’s primary responsibility as family caretaker (p < 0.001), and was also significantly associated with increased odds of testing in the last year (p < 0.01) (data not presented).

**Table 4:** Logistic regression results for HIV testing among women (n = 970) and men (n = 979)

	Women aOR	Men aOR
GEMS (mean score, 23 items) *higher *= *more inequitable*	2.47** (1.46, 4.18)	1.38 (0.95, 2.01)
Norms condoning men’s violence and control over women	1.74* (1.13, 2.69)	1.53* (1.11, 2.10)
Norms around men as the decision-maker in a couple	1.40 (0.97, 2.01)	0.96 (0.77, 1.20)
Norms around men’s toughness and avoidance of help-seeking	1.06 (0.57, 1.98)	0.88 (0.62, 1.24)
Norms around women’s primary responsibility as family caretaker	2.19*** (1.72, 2.80)	1.23 (0.95, 1.60)
Talked with current/most recent sexual partner about getting tested for HIV	1.70* (1.12, 2.59)	1.57** (1.15, 2.14)

Analyses controlled for age, marital status, education; incorporated sampling weights; and accounted for clustering

*p < 0.05, **p < 0.01, ***p < 0.001

Among both men and women, endorsing more inequitable norms around men’s violence and control over women was associated with increased odds of testing. Finally, talking with current/most recent partner about getting tested was associated with increased odds of testing among both women and men.

### Associations with Current Treatment Use

Multivariate analysis results for current ART use, among individuals reporting an HIV-positive status, are presented in Table 5. We found that among women, endorsing more inequitable gender norms (GEM Scale composite) was associated with substantially decreased odds of current ART use (aOR 0.15). Among both women and men, greater endorsement of views around men as the decision-maker in a couple was also associated with substantially decreased odds of current ART use. Among women, endorsement of norms condoning men’s violence and control over women, and around men’s toughness and avoidance of help-seeking, were also associated with decreased odds of ART use. Further, the relatively low sample size for these analyses, particularly among men (n = 48), may have limited the statistical power to find significant associations in some cases.

**Table 5:** Logistic regression results for current antiretroviral treatment (ART) use among women (n = 122) and men (n = 48)

	Women aOR	Men aOR
GEM Scale (mean score, 23 items) *higher *= *more inequitable*	0.15** (0.04, 0.53)	0.57 (0.08, 3.82)
Norms condoning men’s violence and control over women	0.34* (0.12, 0.97)	1.06 (0.21, 5.4)
Norms around men as the decision-maker in a couple	0.18** (0.07, 0.51)	0.28* (0.08, 0.93)
Norms around men’s toughness and avoidance of help-seeking	0.35* (0.13, 0.96)	0.83 (0.17, 4.1)
Norms around women’s primary responsibility as family caretaker	0.54 (0.25, 1.21)	1.30 (0.19, 8.8)

Analyses controlled for age, marital status, and education; incorporated sampling weights; and accounted for clustering

*p < 0.05, **p < 0.01, ***p < 0.001

## Conclusions

A substantial proportion of both men and women in this representative sample of adults in Mpumalanga, South Africa endorsed inequitable gender norms that have been shown to be significantly associated with various negative outcomes. These inequitable norms included agreement that women should tolerate violence and that men should “discipline” their partners, that men should make all major decisions in the relationship, and that women should be solely responsible for caregiving.

The study further demonstrated that respondents living with HIV who more strongly endorsed gender inequity were less likely to be using HIV treatment. This was particularly true for women, with significant associations found with the GEM Scale overall and three out of the four sub-domains (i.e., endorsement of men’s violence and control over women; men as the decision-maker in a couple; and men as reluctant to seek care/help). Among men, a significant association was found specifically around the topic of men as the decision-maker in a couple, whereby men living with HIV who endorsed these inequitable norms were less likely to report current treatment use. These findings support themes raised during earlier (mainly qualitative) studies. For example, in a study with both male and female antiretroviral users in Zimbabwe, some men saw HIV in the family as a threat to their male dignity, and in response, reportedly prevented their partners from accessing care or adhering to ART; and at the same time, many women felt unable to disclose their status to their partners, fearing conflict and violence [35]. A study in South Africa concluded that relationship conflict led to men’s attempt to control their partner’s use of ART [36]. And, in another study with women living with HIV in South Africa, Watt et al. (2017) indicated that a history of sexual trauma hindered women’s HIV care engagement when they were first diagnosed with HIV [37].

Conversely, we found that receiving an HIV test over the past year was in general associated with support for more inequitable norms. This was seen especially among women who endorsed statements around women’s primary role as family caretaker. Several earlier studies had found that substantial family caretaker responsibilities can act as a barrier to accessing services for women, so this result was somewhat unexpected [38–40]. To help explain the finding, we conducted ancillary analyses among respondents, and results suggest that the current association may be accounted for by greater likelihood of testing after having children/during pregnancy. That is, women with children were more likely to endorse norms around women’s primary responsibility as family caretaker (p < 0.001), and they also had increased odds of testing in the last year (p < 0.01). Research with DHS data across 25 countries has similarly found associations between women’s endorsement of inequitable gender norms (i.e., that wife-beating is justified under various circumstances) and early child-bearing [41]. As support for this supposition in South Africa, a study with household-based surveys from a community randomized prevention trial in Soweto found that women who had children under their care were more likely to get tested for HIV [42]. Alternatively, since antenatal services (ANC) are widely available in South Africa and women commonly receive HIV tests in this setting, another explanation could be that women with children are more likely to endorse caretaking norms, and are more likely to have recently tested during ANC, but that the two are not related. It may also be that women endorsing more inequitable norms around men’s power/control in relationships are more likely to test (as we found in the current study) because they are doing so on behalf of their male partners, either because they are directed to do so by their partner or are more concerned about asking their partner to test. This phenomenon—called “proxy testing” [43] —refers to an individual member of a couple (most often a woman) getting tested, with the assumption that the test results will also indicate the status of the partner. This is an incorrect assumption, given couples can be sero-discordant, but it has been periodically reported. Overall, these findings suggest a complex relationship between gender norms and HIV testing, warranting further research and analyses.

Of note, we found that partner communication about testing was important for testing uptake, for both men and women. Specifically, men and women who talked with their most recent/current sexual partner about getting tested for HIV were significantly more likely to have been tested over the last year. Hence, developing partner communication skills, which is also influenced by gender dynamics, is again highlighted here as it has been in other studies [44, 45].

Another interesting finding concerns the topic of men’s avoidance of help-seeking and health care due to considering this a sign of “weakness”/not reflective of a robust male identity. Prior (mainly qualitative) studies had posited this to be a major reason for men’s limited use of services. For example, a study with male and female participants in an ART cohort study in Malawi concluded that men’s masculinity norms inhibited use of treatment until symptoms presented [46]. Other studies with men living with HIV in South Africa found that men’s norms around traditional masculinity prior to positive HIV diagnosis negatively affected their help-seeking behavior and coping with HIV post-diagnosis [47, 48]. And a study with men in Tanzania reported feelings of embarrassment as well as concerns that visiting a clinic for HIV treatment would negatively affect their social status [49]. In the current study, we found quite limited endorsement for this norm. We also did not find associations between this topic and testing and treatment outcomes, with the exception of women who endorsed this norm reporting less ART use. While it is an issue that warrants further research to understand the complexities, it is feasible that this particular factor is not as influential as earlier understood, and interventions promoting HIV service uptake for men should emphasize other factors (such as increasing the convenience of accessing services in places where men gather).

Several limitations to the current study should be highlighted. The data are based on self-report, and this can introduce a fair amount of bias, so future research using other types of more objective measures could strengthen this set of findings. In addition, the limited sample size of HIV-positive individuals, particularly among men (n = 48), meant that we may not have had adequate statistical power for inferential analyses among that subsample (and since we are aware that prevalence rates among men in the community are higher than what was found amongst this sample, those responses may include some bias as well). Further, this is a cross-sectional study, and as such, we cannot make any claims of causation, only association. And finally, there were no variables in the survey to assess whether women received their HIV test via ANC, and thus we were unable to take into account any related confounding influence.

However, this study contributes notably to the field in that it provides quantitative evidence from a community-based sample in South Africa regarding the ways in which views toward gender norms are associated with HIV testing and treatment. Existing evidence suggests that this topic has been explored almost exclusively using qualitative methods. While the current study expands upon or reinforces some of the earlier qualitative findings, it contradicts others. Overall, our findings highlight the point that it is key to understand and address gender norms in interventions to increase uptake of HIV services. Our results also demonstrate the utility of separating out different dimensions of gender norms, since there was not unilateral support for all types of inequitable gender norms, and not all gender norm endorsements were associated in the same way with testing and treatment outcomes.

Given HIV epidemic control goals and South African policy supporting “universal test and start,” evidence is urgently needed to guide choice of strategies and messages to increase HIV service uptake. A clearer understanding of the gender-related barriers to the use of HIV testing and treatment services for both men and women will inform more effective policies and programs. Complexities around HIV testing, for example, need to be taken into account and further investigated to develop appropriate programmatic recommendations. One source of evidence will be this ongoing NIMH-funded community-based trial in Mpumalanga [50, 51], as it will determine the effects of various strategies to shift gender norms and increase use of HIV services.
